# Integrated WGCNA of lncRNA-mRNA Networks Identifies Novel Hub Genes and Potential Therapeutic Agents for Liver Cirrhosis via Molecular Docking Validation

**DOI:** 10.3390/ijms27031260

**Published:** 2026-01-27

**Authors:** Tong Wu, Jiayu Jin, Yuhan Yang, Jing Sui, Yajie Zhou, Hongmei Yuan

**Affiliations:** 1 School of Business Administration, Shenyang Pharmaceutical University, Shenyang 110016, China; 2 Key Laboratory of Environmental Medicine and Engineering of Ministry of Education, Department of Nutrition and Food Hygiene, School of Public Health, Southeast University, Nanjing 210009, China

**Keywords:** liver cirrhosis, long non-coding RNAs, weighted gene co-expression network analysis, drug target, molecular docking

## Abstract

Liver cirrhosis (LC) is a complex pathological condition characterized by extensive transcriptomic reprogramming, yet the regulatory role of non-coding RNAs in disease progression remains poorly understood. This study aimed to systematically investigate long non-coding RNA (lncRNA)-messenger RNA (mRNA) interaction networks in LC through weighted gene co-expression network analysis (WGCNA). Gene expression profiles from datasets GSE197406, GSE107170, and GSE17548 were retrieved from the Gene Expression Omnibus (GEO) database, and differentially expressed lncRNAs and mRNAs were identified. Co-expression modules were constructed using WGCNA. Furthermore, functional enrichment analyses were conducted and drug repurposing opportunities were evaluated. Additionally, lncRNA-mRNA co-expression networks and lncRNA-mRNA-pathway networks were constructed to identify key regulatory relationships. Molecular docking simulations were subsequently performed to validate potential drug–target interactions. The results revealed several co-expression modules significantly associated with LC, particularly the turquoise module (r = 0.81). Genes within this module were enriched in several biological pathways, including the PI3K-Akt signaling pathway, NF-κB signaling pathway, and chemokine signaling pathway. The hub lncRNA in the turquoise module, *NONHSAT134945.2*, was found to be co-expressed with mRNAs involved in inflammasome-mediated pyroptosis and hepatocyte activation, such as *CSF1R*, *HCK*, and *CASP1*. Based on this hub gene signature, AB-1010, GW768505A, and Dasatinib were identified as potential therapeutic candidates. Molecular docking analysis confirmed that these compounds exhibit high binding affinity to CSF1R and HCK, with all interatomic distances maintained below 3.5 Å. These findings provide new insights into the molecular mechanisms underlying LC and suggest that the *NONHSAT134945.2*–*CSF1R*/*HCK* axis may serve as a valuable target for future translational research and therapeutic development.

## 1. Introduction

Liver cirrhosis (LC) represents the irreversible and advanced stage of nearly all chronic liver diseases, posing a significant global health burden due to its high morbidity and mortality rates [1,2]. Pathologically, LC is characterized by the progressive replacement of functional hepatic parenchyma with diffuse fibrosis, excessive extracellular matrix deposition, and the development of regenerative nodules, resulting in the disruption of normal hepatic architecture and impaired liver function [3]. This structural and functional deterioration leads to severe clinical consequences, including portal hypertension, hepatic failure, and a substantially increased risk of hepatocellular carcinoma (HCC) [4]. Although the primary etiologies of LC are well-established, effective anti-fibrotic therapies capable of reversing established cirrhosis remain critically limited [5]. Therefore, a comprehensive understanding of the underlying molecular mechanisms driving the progression from chronic liver injury to established cirrhosis, along with the identification of novel pharmacological targets and potentially repurposable therapeutic agents, is an urgent priority in translational hepatology.

Emerging evidence underscores the critical roles of the transcriptome, particularly long non-coding RNAs (lncRNAs) and messenger RNAs (mRNAs), in the pathogenesis and progression of hepatic fibrosis and cirrhosis [6,7]. LncRNAs, defined as transcripts exceeding 200 nucleotides without protein-coding potential, function as sophisticated regulators within the complex landscape of gene expression [8]. Their regulatory mechanisms are highly diverse and include processes such as chromatin remodeling and transcriptional interference [9]. Dysregulated lncRNA-mRNA interactions are mechanistically implicated in the key stages of LC progression, including hepatic stellate cell (HSC) activation, inflammatory signaling, and excessive extracellular matrix (ECM) accumulation [10]. However, the majority of existing studies have primarily relied on univariate or single-gene differential expression analysis. This conventional approach often fails to capture the systemic complexity, coordinated gene fluctuations, and module-level organization of genes that collectively drive the cirrhotic phenotype.

To overcome the aforementioned limitations inherent in single-gene analysis, Weighted Gene Co-expression Network Analysis (WGCNA) provides a robust systems biology framework for inferring complex regulatory relationships from high-throughput transcriptomic data [11]. Distinct from conventional differential expression studies, WGCNA identifies highly interconnected gene clusters (modules) based on the topological overlap of their expression patterns [12]. Crucially, WGCNA correlates these identified modules with external clinical traits, thereby enabling the identification of key functional modules and their associated hub genes that are hypothesized to act as central drivers of the disease-associated regulatory network [13]. Although WGCNA has been successfully applied to decipher the molecular architecture of various complex diseases, including numerous cancers and metabolic disorders, its comprehensive and integrated application in systematically analyzing the specific lncRNA-mRNA co-expression landscape driving the pathogenesis of LC remains underexplored and warrants further investigation.

Furthermore, the identification of potential biomarkers represents only the initial step; translating these findings into effective therapeutic interventions is crucial. Drug repurposing, defined as the strategy of identifying novel indications for existing approved or investigational drugs, offers a cost-effective and time-efficient alternative to conventional drug discovery [14]. Nevertheless, few studies have systematically integrated WGCNA-based hub gene identification with small-molecule drug prediction in the context of LC. Moreover, computational validation techniques, such as molecular docking, are essential to predict the binding affinity and interaction stability between candidate drugs and their protein targets, thereby providing in silico evidence for therapeutic efficacy [15].

In the present study, we adopted an integrated bioinformatics framework to construct a co-expression network of lncRNAs and mRNAs in LC using WGCNA. The aim was to identify key functional modules and hub genes significantly associated with the disease status. Beyond network construction, we utilized the identified hub genes to predict potential therapeutic agents and validated the interactions between the top candidate drugs and core targets through molecular docking. This multi-step approach not only provides novel insights into the molecular architecture of LC but also identifies promising small-molecule compounds for its treatment.

## 2. Results

### 2.1. Global Differentially Gene Expression Patterns in Liver Tissue

**Figure 1:**
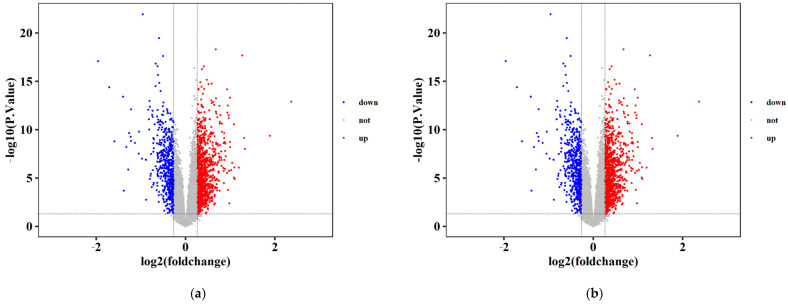
Differential expression analysis of mRNAs and lncRNAs in liver condition. (**a**) mRNAs: liver cirrhosis vs. control; (**b**) lncRNAs: liver cirrhosis vs. control.

A total of 1933 differentially expressed genes (DEGs), comprising 333 lncRNAs and 1600 mRNAs, were identified between LC and normal control tissues based on the predefined criteria (*p* < 0.05 and fold change (FC) > 2 or <0.5). Among these, 746 genes were significantly downregulated (187 lncRNAs and 559 mRNAs), while 1187 genes were significantly upregulated (146 lncRNAs and 1041 mRNAs) (Figure 1).

### 2.2. GO and KEGG Pathway Enrichment Analysis of DEGs

**Figure 2:**
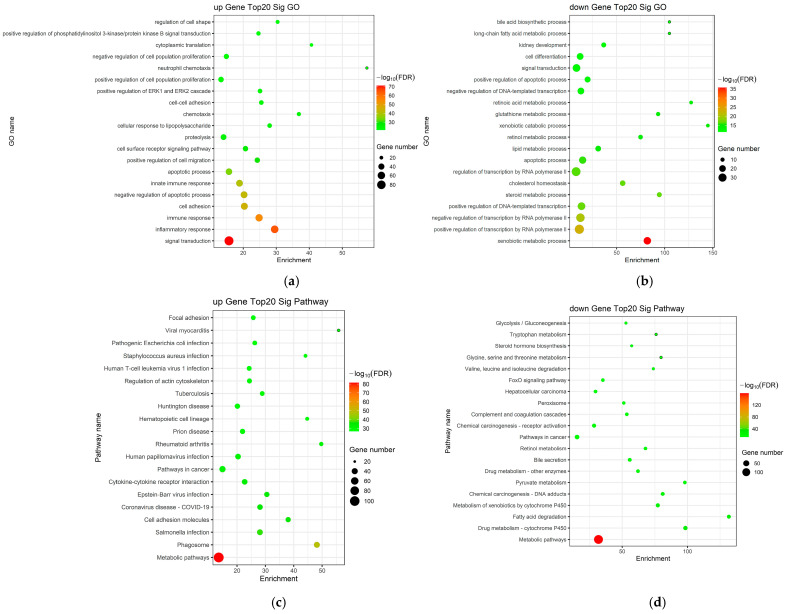
Functional enrichment analysis in the DEGs. (**a**,**b**) GO enrichment analysis of up-regulated and down-regulated mRNAs; (**c**,**d**) KEGG pathway enrichment analysis of up-regulated and down-regulated mRNAs.

To further elucidate the biological significance of the identified DEGs in LC, Gene Ontology (GO) and Kyoto Encyclopedia of Genes and Genomes (KEGG) pathway enrichment analyses were performed. GO analysis revealed that these DEGs were significantly enriched in biological processes (BP) related to collagen metabolic process, inflammatory response, extracellular matrix organization, and angiogenesis. In terms of cellular components (CC), the DEGs were primarily associated with the extracellular matrix, collagen-containing extracellular matrix, endoplasmic reticulum, and focal adhesion. Regarding molecular functions (MF), the DEGs exhibited significant enrichment in cytokine activity, integrin binding, growth factor activity, and protease inhibitor activity (Figure 2a,b).

Furthermore, KEGG pathway analysis was conducted to identify the key signaling pathways involved in the progression of LC. The results revealed that the DEGs were predominantly integrated into several critical pathways, including the TGF-β signaling pathway, PI3K-Akt signaling pathway, NF-κB signaling pathway, ECM–receptor interaction, and HIF-1 signaling pathway (Figure 2c,d). These enriched pathways and functional categories provide essential insights into the molecular cascades and pathological mechanisms underlying the development of LC.

### 2.3. Construction of the Gene Co-Expression Network

**Figure 3:**
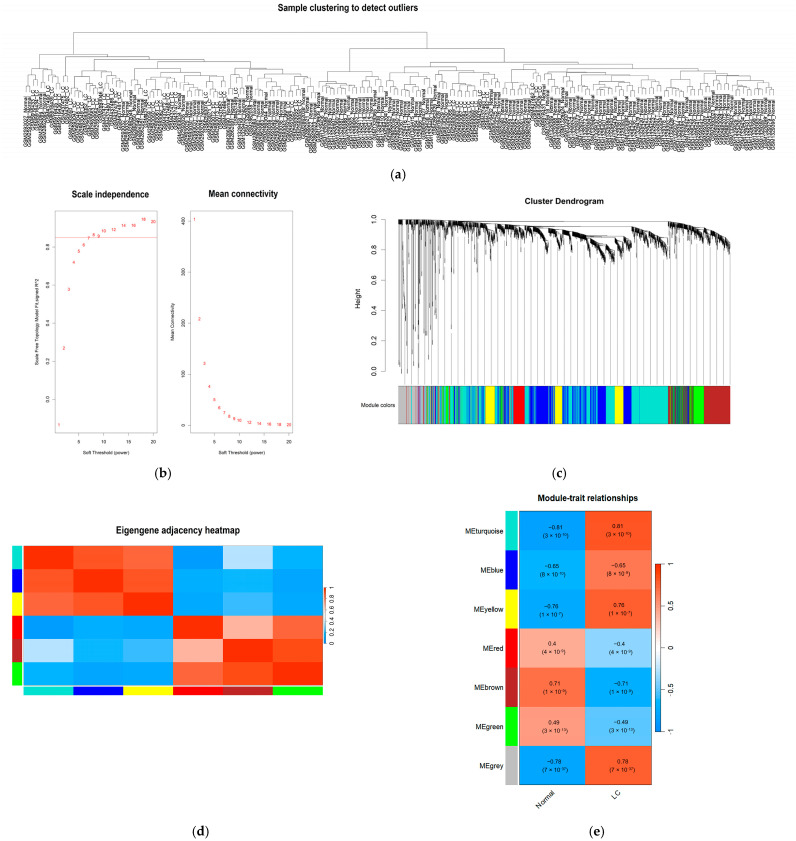
WGCNA was used to identify co-expression modules among different expression genes. (**a**) no outlier samples were identified based on the cut-off height; (**b**) the soft threshold power; (**c**) clustering dendrograms of WGCNA (derived from the dynamic tree cutting method the colorful bands facilitate direct visual module allocation contrasts); (**d**) heatmap of multiple gene modules illustrating; (**e**) the correlation of genes of modularity and liver cirrhosis (each cell contains the correlation coefficient and the value of *p*).

Hierarchical clustering was initially performed on the GSE197406, GSE107170, and GSE17548 datasets to identify potential outlier samples. The cluster analysis confirmed the absence of any outlier samples among the 57 LC and 144 normal control samples (Figure 3a). To ensure a comprehensive expression profile, a total of 333 lncRNAs and 1600 mRNAs were included in the network construction.

The evaluation of scale independence and mean connectivity determined an optimal soft-thresholding power (β) of 7 (Figure 3b). Based on this β value, hierarchical clustering dendrograms were constructed to identify co-expression modules through the dynamic tree-cutting algorithm. A total of 7 distinct modules were identified, including turquoise, blue, yellow, red, brown, green, and grey (Figure 3c). The specific distribution of lncRNAs and mRNAs within each module is summarized in Table 1. Furthermore, the inter-module relationships were visualized using an eigengene adjacency heatmap, which illustrates the connectivity and independence among the identified modules (Figure 3d).

### 2.4. Identification of Clinically Significant Modules

To correlate the gene modules with the pathological status of LC, we calculated the correlation between the module eigengenes and the clinical trait (cirrhosis vs. normal). The analysis revealed that the turquoise module exhibited the strongest positive correlation with cirrhosis status (r = 0.81), with high statistical significance (*p* < 3 × 10^−10^) (Figure 3e). This module contained 640 genes, including 93 lncRNAs and 547 mRNAs, and was designated as the key module for subsequent functional analysis and hub gene identification (Table 1).

### 2.5. Functional Enrichment Analysis of the Key Module

**Figure 4:**
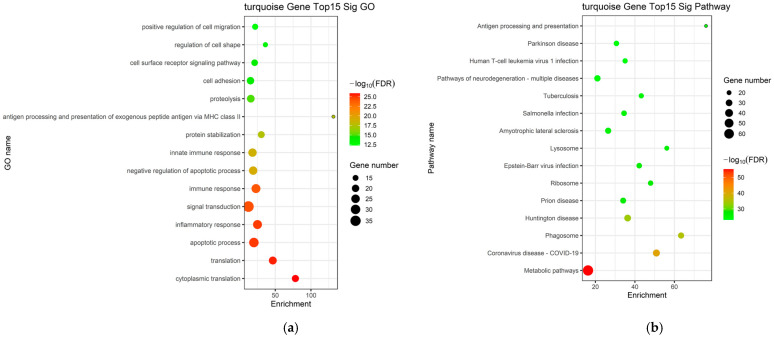
Functional enrichment evaluation in the blue and turquoise modules. (**a**) GO enrichment analysis of mRNAs within the turquoise modules; (**b**) KEGG pathway enrichment analysis of mRNAs within the d turquoise modules.

Genes within the key turquoise module were subjected to GO and KEGG pathway enrichment analyses. The GO enrichment analysis predominantly identified BP associated with activation and migration of hepatic stellate cells, persistent inflammatory responses, and the regulation of apoptotic pathways, collectively elucidating the complex cellular dynamics underlying the progression of LC (Figure 4a). The identified pathways were closely related to pro-fibrotic drive, inflammatory perpetuation, and metabolic-hypoxic adaptation, including the TGF-β signaling pathway, PI3K-Akt signaling pathway, focal adhesion, chemokine signaling pathway, cytokine–cytokine receptor interaction, NF-κB signaling pathway, HIF-1 signaling pathway, and metabolic pathways (Figure 4b).

### 2.6. Construction of lncRNA-mRNA-Nets and lncRNA-mRNA-Pathway-Nets

**Figure 5:**
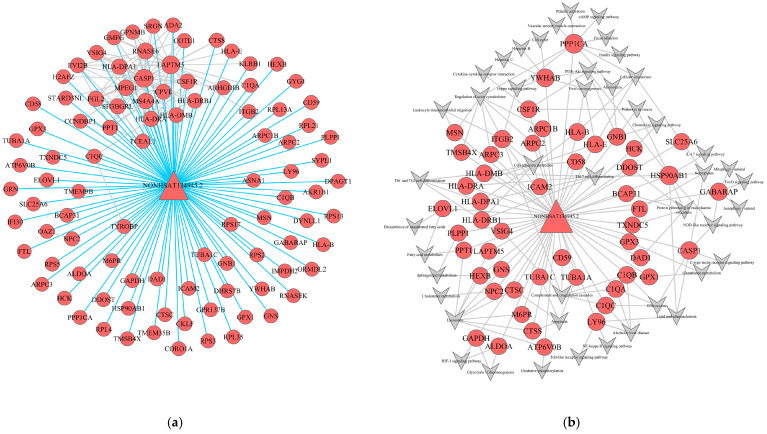
lncRNA-mRNA-nets and LncRNA-mRNA-pathway-nets of hub Genesin turquoise module. (**a**,**b**) lncRNA-mRNA-nets of turquoise module; (**b**) LncRNA-mRNA-pathway-nets of turquoise module. (Triangles as lncRNAs, circles as mRNAs. The size reflects the regulatory ability).

To further elucidate the functional roles of lncRNAs within the turquoise module, a localized lncRNA-mRNA co-expression network was constructed. A total of one lncRNA and 97 mRNAs were identified as hub nodes within this specific sub-network (Figure 5a). The network architecture revealed a complex regulatory crosstalk between non-coding and coding transcripts, where a single lncRNA was strongly correlated with multiple mRNAs. This highly connected topology suggests that these lncRNAs may exert broad regulatory influences over their associated mRNAs, enabling functional inference of the lncRNAs based on the well-established biological roles of their mRNA partners.

Furthermore, to explore the specific mechanisms by which lncRNAs modulate signaling cascades, we integrated the co-expression data with enriched pathways to construct a lncRNA-mRNA-Pathway interaction network. Within this network, the turquoise module comprised one lncRNA and 51 mRNAs associated with key pathological processes (Figure 5b). The lncRNA *NONHSAT134945.2* exhibited significant co-expression with these 51 mRNAs, including critical effectors such as *PPP1CA*, *CASP1*, *HCK*, *CSF1R*, *HSP90AB1*, and *CD59*. Functional mapping indicated that this lncRNA-mediated network was predominantly enriched in pathways vital to LC progression, including the PI3K-Akt signaling pathway, NF-κB signaling pathway, focal adhesion, HIF-1 signaling, and apoptosis. These findings suggest that *NONHSAT134945.2* may function as a master regulator in the turquoise module, potentially driving the cirrhotic phenotype through the integrated modulation of inflammatory and fibrogenic signaling axes.

### 2.7. Identification of Key Hub Proteins Through Protein–Protein Interaction Network Analysis

**Figure 6:**
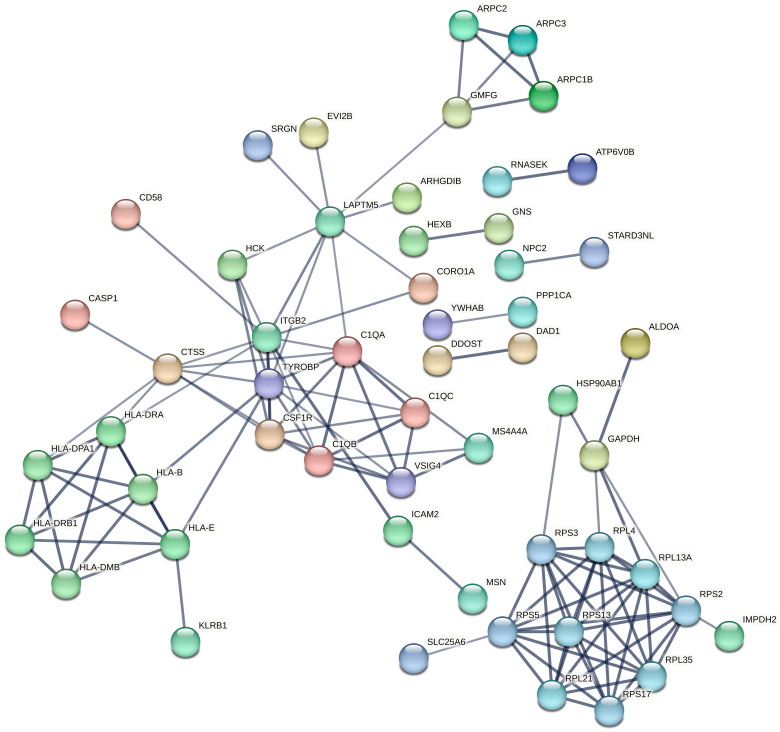
Protein–protein interaction (PPI) networks of the turquoise modules. (The thickness of the line represents edge confidence, with thicker lines indicating a higher confidence level).

To further explore the functional interactions among the candidate genes from the turquoise module after constructing the lncRNA-mRNA-nets, a protein–protein interaction (PPI) network was constructed using the STRING database (Figure 6). The network comprised 96 nodes and 120 edges, with an average node degree of 2.5. The PPI enrichment *p*-value was less than 1.0 × 10^−16^, indicating that the proteins exhibited significantly more interactions among themselves than would be expected by chance, thereby supporting substantial biological connectivity.

### 2.8. Identification of Potential Therapeutic Candidates for Liver Cirrhosis

The mRNA component of the core hub genes was used as a signature query against the Drug Signatures Database (DSigDB) for drug repurposing. The screening successfully identified 379 small-molecule compounds with significantly negative enrichment scores (*p* < 0.05), indicating a predicted ability to reverse the LC-associated gene expression signature (Table 2). The compound with the highest predicted therapeutic potential was AB-1010 Kinome Scan (Combined Score = 1573.5), followed by GW768505A GSK (Combined Score = 1170.5) and Dasatinib RBC (Combined Score = 699.2), collectively targeting the hub kinases CSF1R and HCK. These candidates primarily belong to the class of tyrosine kinase inhibitors.

### 2.9. Molecular Docking Validation

**Figure 7:**
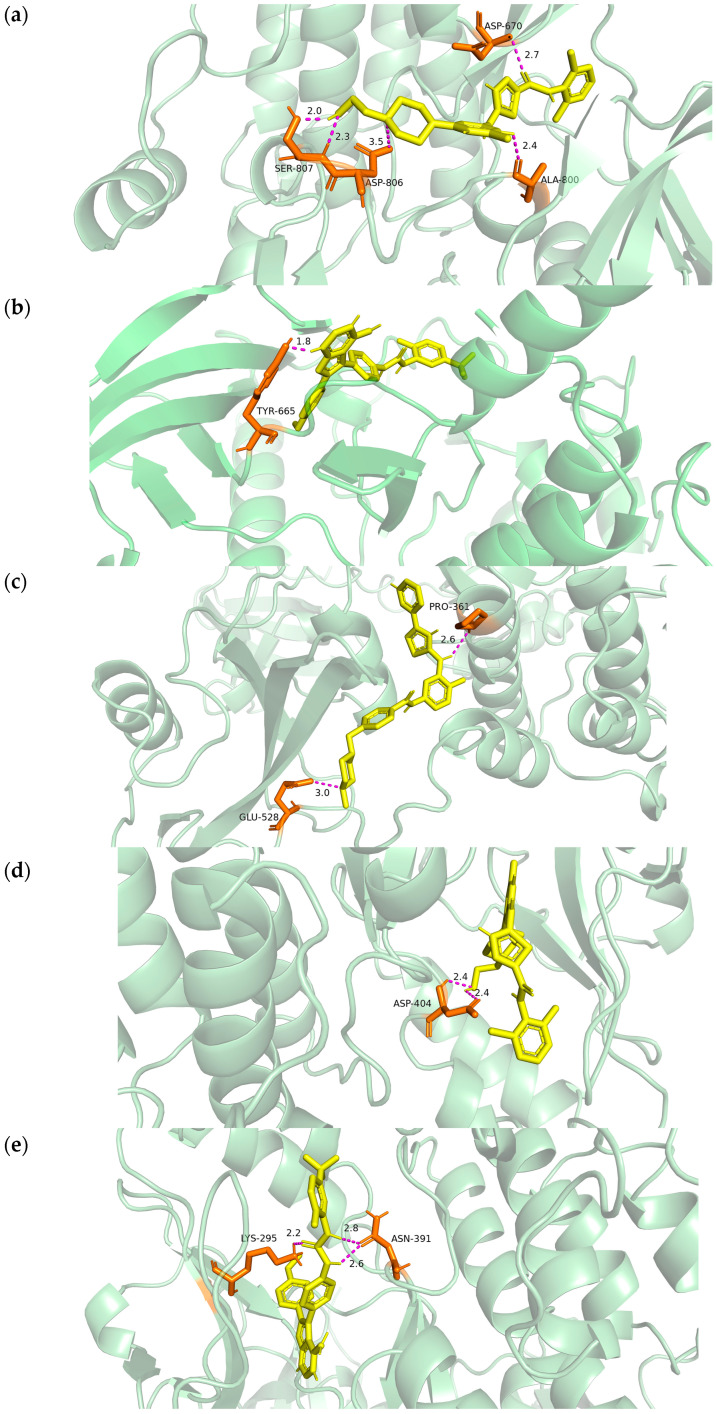
Docking results of available proteins small molecules. (**a**) Molecular docking of CSF1R to Dasatinib and the hydrogen binding sites located on the amino acids of SER-807, ASP-806, ASP-670 and ALA-800; (**b**) molecular docking of CSF1R to GW768505A and the hydrogen binding sites located on the amino acids of TYR-665; (**c**) molecular docking of HCK to AB1010 and the hydrogen binding sites located on the amino acid of PRO-361 and GLU-528; (**d**) molecular docking of HCK to GW768505A and the hydrogen binding sites located on the amino acid of LYS-295 and ASN-391; (**e**) molecular docking of HCK to Dasatinib and the hydrogen binding sites located on the amino acid of ASP-404.

To evaluate target druggability, we prioritized the interaction architectures of CSF1R (PDB ID: 8JOT) and HCK (PDB ID: 9BYJ), the crystal structures of which were obtained from the RCSB Protein Data Bank [16]. Visualization confirmed that AB1010, GW768505A, and Dasatinib occupied the catalytic pocket of CSF1R with a binding affinity of −9.2 kcal/mol, −9.2 kcal/mol and −10.6 kcal/mol, respectively. The hydrogen binding sites were located at the amino acids of SER-807, ASP-806, ASP-670 and ALA-800 (for Dasatinib) and TYR-665 (for GW768505A) (Figure 7a,b). The binding energy of HCK, AB1010, GW768505A, and Dasatinib was −10.1 kcal/mol, −9.4 kcal/mol and −11.9 kcal/mol, respectively. Meanwhile, the hydrogen bonding sites were located at the amino acids of PRO-361, GLU-528 (for AB1010), LYS-295, ASN-391 (for GW768505A), and ASP-404 (for Dasatinib) (Figure 7c–e). All interatomic distances remained within the strict 3.5Å threshold, supporting the selection of these compounds as high-fidelity candidates for LC therapy.

## 3. Discussion

LC is a multifaceted pathological condition characterized by the progressive architectural distortion of the liver [17]. Although conventional differential expression analysis can detect individual gene expression changes, it often fails to capture the synergistic interactions within the transcriptome. In this study, we utilized an integrated systems biology approach, combining WGCNA with PPI network analysis, to investigate the lncRNA-mRNA co-expression networks associated with LC. Our results identified the turquoise module as the most critical functional module, exhibiting a robust correlation (r = 0.81) with the cirrhotic phenotype. This module represents a core set of molecular perturbations underlying hepatic architectural deterioration, providing a targeted framework for identifying novel therapeutic targets and candidate small molecules.

The functional architecture of the turquoise module delineates a sophisticated pathological network underlying LC progression. At the signaling interface, the robust enrichment of cytokine–cytokine receptor interaction and chemokine signaling pathways indicates a high-capacity system for inflammatory cell recruitment [18,19]. These upstream pathways provide the sustained stimuli required for the activation of the NF-κB and PI3K-Akt signaling pathways, which function as central regulators of HSC transdifferentiation [20,21]. While NF-κB signaling pathway perpetuates the pro-inflammatory microenvironment [22], the PI3K-Akt signaling pathway confers a crucial survival advantage to activated myofibroblasts, ensuring the persistence of the fibrotic drive [23].

Furthermore, the involvement of HIF-1 signaling and metabolic pathways indicates that the cirrhotic liver undergoes systemic reprogramming to adapt to localized hypoxia [24,25]. Collectively, the turquoise module represents not just a collection of co-expressed genes, but a synchronized program where the persistence of inflammation and metabolic-hypoxic adaptation converge to stabilize hepatic architectural collapse.

Network analysis identifies *NONHSAT134945.2* as a pivotal regulatory lncRNA in LC, exhibiting high-degree centrality with 51 mRNA partners. Its pathological relevance is supported by significant correlations with *CSF1R* and *HCK*. Specifically, *CSF1R* orchestrates the recruitment and pro-fibrogenic polarization of monocyte-derived macrophages [26], while *HCK* functions as a critical transducer of signaling fluxes driving hepatocytes’ activation and survival [27,28]. Notably, *NONHSAT134945.2* has not been previously characterized in the literature. Its identification as a pivotal hub gene in our network, combined with its high-degree connectivity to pro-fibrotic drivers like *CSF1R* and *HCK*, highlights it as a novel candidate for future experimental validation in hepatic stellate cell activation models. Moreover, the association between *NONHSAT134945.2* and *CASP1* suggests a master regulatory role in sustaining the pro-inflammatory microenvironment [29]. Furthermore, it shows significant correlations with *LAPTM5* and *GNB1*, which are involved in lysosomal membrane permeabilization and G-protein coupled receptor signaling, respectively [30,31].

To address the scarcity of anti-fibrotic agents [32], we leveraged the turquoise module signature to identify candidates capable of global transcriptomic reversal. Our screening identified AB-1010, GW768505A, and dasatinib as top candidates, all of which function as tyrosine kinase inhibitors (TKIs). This selection is biologically consistent with the requirement of aberrant phosphorylation for HSC activation [33,34,35]. Notably, these candidates exhibit a high degree of pharmacological convergence by collectively targeting the hub kinases CSF1R and HCK, which are pivotal in mediating the inflammatory-fibrotic crosstalk [36,37]. By modulating multiple nodes within the PI3K-Akt, cytokine–cytokine receptor interaction, and chemokine signaling pathway, these repositioned drugs offer a systemic therapeutic intervention that aligns with the polygenic complexity of cirrhosis.

The translational feasibility of the identified TKIs, such as AB-1010, GW768505A, and dasatinib, warrants careful consideration given the compromised hepatic reserve in cirrhotic patients. Although TKIs have demonstrated potent anti-fibrotic potential by blocking PDGFR and VEGFR signaling pathways in activated HSCs, their systemic application is often limited by hepatotoxicity [38,39,40]. To overcome these limitations, future research should focus on structural optimization to enhance liver-specific targeting or the utilization of advanced drug delivery systems. For instance, nanoparticle-mediated delivery specifically targeting HSC surface markers could significantly minimize systemic toxicity while maximizing therapeutic concentrations at the site of fibrosis [41]. Our computational findings provide a prioritized list of chemical scaffolds that could serve as templates for developing next-generation, liver-safe anti-fibrotic agents.

Molecular docking anchored our transcriptomic predictions in biochemical reality by resolving the interactions with CSF1R (8JOT) and HCK (9BYJ) kinases central to LC progress [42,43]. The simulation revealed an exceptionally stable energetic profile, with binding affinities for AB-1010, GW768505A, and dasatinib reaching from −9.2 to −11.9 kcal/mol. Specifically, the CSF1R complex is stabilized by a coordinated hydrogen-bonding network involving SER-807, ASP-806, and ALA-800, while HCK engagement is reinforced by critical anchoring interactions at PRO-361 and GLU-528. The consistent interatomic distances, all maintained below the 3.5Å threshold, confirm high structural complementarity and the formation of stable hydrogen-bonding networks [44].

Despite the rigor of our bioinformatics framework, certain limitations should be considered. Due to the limitations of public database, specific clinical characteristics such as etiology and disease stage could not be fully stratified. However, the identified hub genes represent a convergent molecular signature of liver cirrhosis, offering insights into the universal mechanisms of hepatic architectural distortion. Our analysis relied on retrospective data from public repositories, which may be subject to platform heterogeneity. Furthermore, although molecular docking provides a structural basis for drug action, the therapeutic efficacy of AB-1010, GW768505A, and dasatinib, as well as the regulatory role of NONHSAT134945.2, requires validation through in vitro assays in activated HSCs and in vivo studies in animal models of cirrhosis. Future research focused on the specific mechanism by which NONHSAT134945.2 modulates its downstream mRNA targets will be crucial for clarifying its potential as a therapeutic target.

## 4. Materials and Methods

### 4.1. Data Acquisition and Preprocessing

Gene expression datasets related to LC were retrieved from the Gene Expression Omnibus (GEO) database (https://www.ncbi.nlm.nih.gov/geo/ (accessed on 20 November 2025)) [45]. The datasets (GSE197406, GSE107170, and GSE17548), derived from GPL570 platform, were selected for this study. These datasets comprise 57 LC tissue samples and 144 normal liver tissue samples.

**Figure 8:**
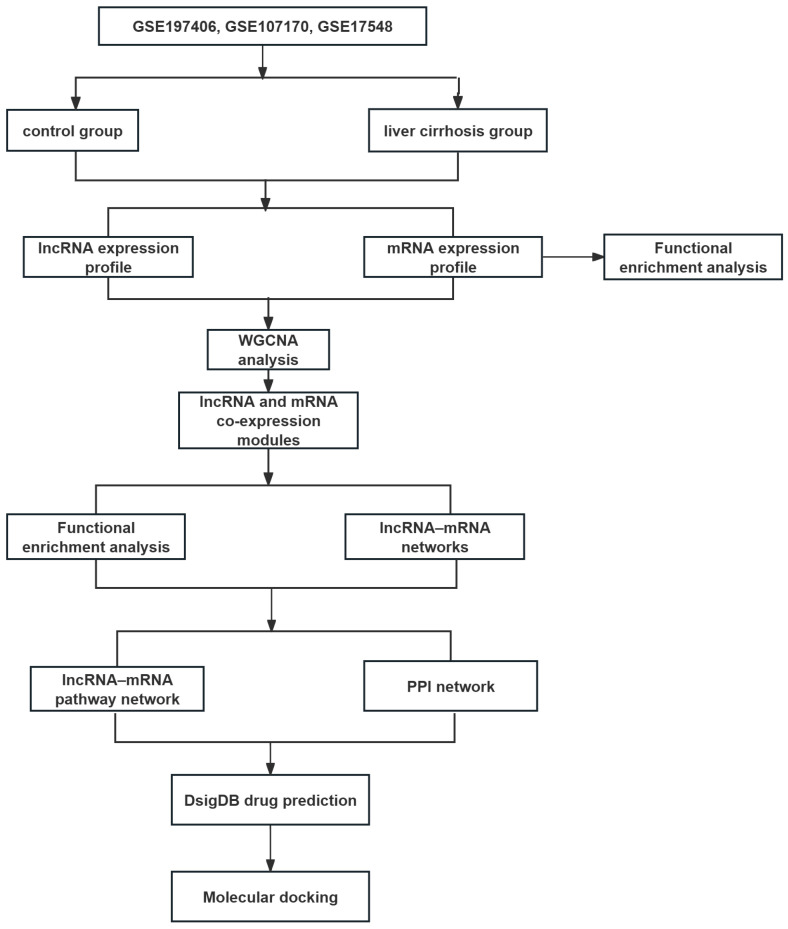
Overview of the study design.

Raw data were normalized using the limma package in R software (ersion 4.5.2; R Foundation for Statistical Computing, Vienna, Austria). According to the shared genes, datasets were merged and normalized to remove the batch effect between arrays by the “SVA” packages in R software [46] (Figure S1). To identify key genes associated with LC, DEGs were screened. To ensure the robustness of the differential expression analysis, *p*-values were adjusted for multiple testing using the Benjamini–Hochberg False Discovery Rate (FDR) method. Meanwhile, genes with an adjusted *p* < 0.05 and FC > 2 or <0.5 were considered significantly differentially expressed. Only lncRNAs and mRNAs meeting the significance thresholds were selected for the subsequent WGCNA. A flow chart of this study is presented in Figure 8.

### 4.2. Construction of Weighted Gene Co-Expression Network

The weighted gene co-expression network was constructed using the WGCNA package in R to identify functional gene modules. First, a similarity matrix was constructed by calculating the Pearson correlation coefficient between all gene pairs. To ensure the network satisfied the scale-free topology criterion, an appropriate soft-thresholding power (β) was selected using the pickSoftThreshold function. For the construction of the weighted co-expression network, the soft-thresholding power (β) was determined by the first power that reached a scale-free topology fit index (R^2^) of 0.80, ensuring a balance between network connectivity and biological relevance.

Subsequently, the adjacency matrix was transformed into a topological overlap matrix (TOM) to measure the network connectivity of the genes, and the corresponding dissimilarity (1-TOM) was calculated. Hierarchical clustering was performed based on 1-TOM to construct a clustering tree. Gene modules were identified using the Dynamic Tree Cut algorithm with a minimum module size of 30 genes. Modules exhibiting highly similar expression profiles (dissimilarity of module eigengenes <0.25) were merged.

To identify the key functional modules associated with LC, we evaluated candidate modules based on statistical significance and network capacity. Instead of solely relying on correlation coefficients, modules were prioritized and selected based on a significance threshold of *p* < 0.05, in conjunction with the abundance of lncRNAs and mRNAs contained within them. This selection strategy ensures that the identified modules are not only statistically significant but also possess sufficiently complex regulatory networks to contribute to the pathological progression of LC.

### 4.3. Functional Enrichment Analysis

To explore the biological functions of genes, GO enrichment analysis (including Biological Process, Cellular Component, and Molecular Function) [47] and KEGG pathway analysis [48] were conducted using the clusterProfiler package in R [49]. A significance threshold of adjusted *p* < 0.05 was applied to identify biological terms and pathways.

Beyond standard functional categorization, this analysis served as a pivotal bridge between the abstract co-expression clusters and the actual pathological architecture of LC. By mapping the turquoise module’s components to high-level signaling cascades, we were able to characterize a synchronized transcriptomic landscape where inflammatory recruitment and metabolic-hypoxic adaptation converge. This integrative strategy represents a primary achievement of our work, shifting the focus from isolated gene changes to a comprehensive understanding of the synergistic interaction networks that drive hepatic architectural collapse. This approach not only confirmed the known pro-fibrotic pathways but also facilitated the discovery of novel regulatory axes involving previously uncharacterized lncRNAs.

### 4.4. Protein–Protein Interaction Network Construction

To further refine the key drivers, a PPI network was constructed using the STRING database (https://string-db.org/) with a high confidence score threshold of >0.7 [50].

### 4.5. Prediction of Potential Therapeutic Agents

To evaluate the pharmacological tractability of the identified hub genes, a systematic drug–target interaction screen was performed. The core gene signatures were queried against the DSigDB (https://dsigdb.tanlab.org/DSigDBv1.0/ (accessed on 28 November 2025)), an expansive repository correlating small-molecule compounds with their transcriptional targets [51]. By mapping upregulated and downregulated hub genes to the database’s chemical-gene sets, potential therapeutic candidates were predicted.

### 4.6. Molecular Docking Verification

Based on the drug candidates predicted via DSigDB, 3D structures were curated from PubChem (https://pubchem.ncbi.nlm.nih.gov/) [52]. For compounds lacking verified 3D coordinates, the next most significant candidates were sequentially selected according to their *p*-value ranking. To ensure conformational stability for molecular docking, all ligand geometries were subjected to energy minimization via ChemBioOffice [53]. Receptor protein structures were concurrently retrieved from UniProt-linked databases (https://www.uniprot.org/) to facilitate atomic-level interaction modeling [54].

Molecular docking simulations were performed using AutoDock Vina (version 1.1.2) [55]. Receptor preparation involved the removal of water molecules and heteroatoms, followed by the addition of polar hydrogen atoms and Gasteiger charges using AutoDockTools. The docking parameters included a grid box centered on the known active binding site of the target kinases, with a spacing of 0.375 Å and a dimension of 20 × 20 × 20 Å^3^. Binding affinities were calculated based on the lowest energy conformations, and a binding energy threshold of <−5.0 kcal/mol was applied to identify stable interactions. The final docking results and ligand–receptor interactions were visualized using PyMOL (version 2.5; Schrödinger, LLC, New York, NY, USA) [56].

### 4.7. Statistical Analysis

All statistical analyses were conducted using R software (version 4.5.2). Differential expression analysis was performed using Student’s *t*-test. A *p* < 0.05 was considered statistically significant unless otherwise specified.

## 5. Conclusions

In conclusion, this study provides a comprehensive map of the lncRNA-mRNA co-expression network in LC. We identified the turquoise module and the hub lncRNA NONHSAT134945.2 as central drivers of disease progression. By integrating transcriptomic mining with structural bioinformatics, we proposed AB-1010, GW768505A, and dasatinib as novel candidates for LC therapy. These findings elucidate the complex regulatory circuitry of the non-coding landscape in LC, while establishing a high-confidence framework for target prioritization and subsequent translational intervention.

## Figures and Tables

**Table 1:** The number of mRNAs and lncRNAs across the 7 modules.

Module	All Numbers	mRNA Numbers	lncRNA Numbers
turquoise	640	547	93
blue	422	422	0
brown	235	207	28
yellow	189	145	44
grey	166	96	70
green	163	146	17
red	79	37	42

**Table 2:** LC-related biomarkers and parts of targeted drugs.

Term	*p* Value	Odds Ratio	Combined Score	Genes
AB-1010 Kinome Scan	9.89758 × 10^−5^	170.6581197	1573.576348	CSF1R; HCK
GW768505A GSK	0.00015594	133.5250836	1170.486231	CSF1R; HCK
carnosol CTD 00002718	0.00015594	133.5250836	1170.486231	GAPDH; PPP1CA
Toxoflavin TTD 00011503	7.39307 × 10^−7^	76.13397129	1074.825391	HSP90AB1; ITGB2; GAPDH; PPP1CA
Gly-His-Lys PC3 UP	0.00024108	105.867374	881.9155959	HSP90AB1; GAPDH
Cobalt sulfate CTD 00001238	0.000257041	102.3333333	845.9155332	ALDOA; GAPDH
Dasatinib RBC	0.000344393	87.69230769	699.2344145	CSF1R; HCK

## Data Availability

The original data presented in the study are openly available in the GEO database at https://www.ncbi.nlm.nih.gov/geo/query/acc.cgi?acc=GSE197406 (accessed on 20 November 2025), accession numbers: GSE197406, GSE107170, and GSE17548.

## Supplementary Materials

The following supporting information can be downloaded at https://www.mdpi.com/article/10.3390/ijms27031260/s1.

## Abbreviations

The following abbreviations are used in this manuscript:

LC	Liver cirrhosis
WGCNA	Weighted gene co-expression network analysis
GEO	Gene Expression Omnibus
lncRNAs	Long non-coding RNAs
mRNAs	Messenger RNAs
HSC	hepatic stellate cell
BP	Biological processes
CC	Cellular components
MF	Molecular functions
DEGs	differentially expressed genes
GO	Gene Ontology
KEGG	Kyoto Encyclopedia of Genes and Genomes
PPI	Protein–Protein Interaction
DSigDB	Drug Signatures Database
TKIs	Tyrosine kinase inhibitors
FDR	False discovery rate
FC	Fold change
TOM	Topological overlap matrix
