# Supplementary material for: Integrated WGCNA of lncRNA-mRNA Networks Identifies Novel Hub Genes and Potential Therapeutic Agents for Liver Cirrhosis via Molecular Docking Validation
Source: Int J Mol Sci. 2026 Jan 27;27(3):1260. doi: 10.3390/ijms27031260 (PMC12898157; doi:10.3390/ijms27031260)
Supplement: Supplementary file 1 [file ijms-27-01260-s001.zip › ijms-4108813-supplementary.pdf]

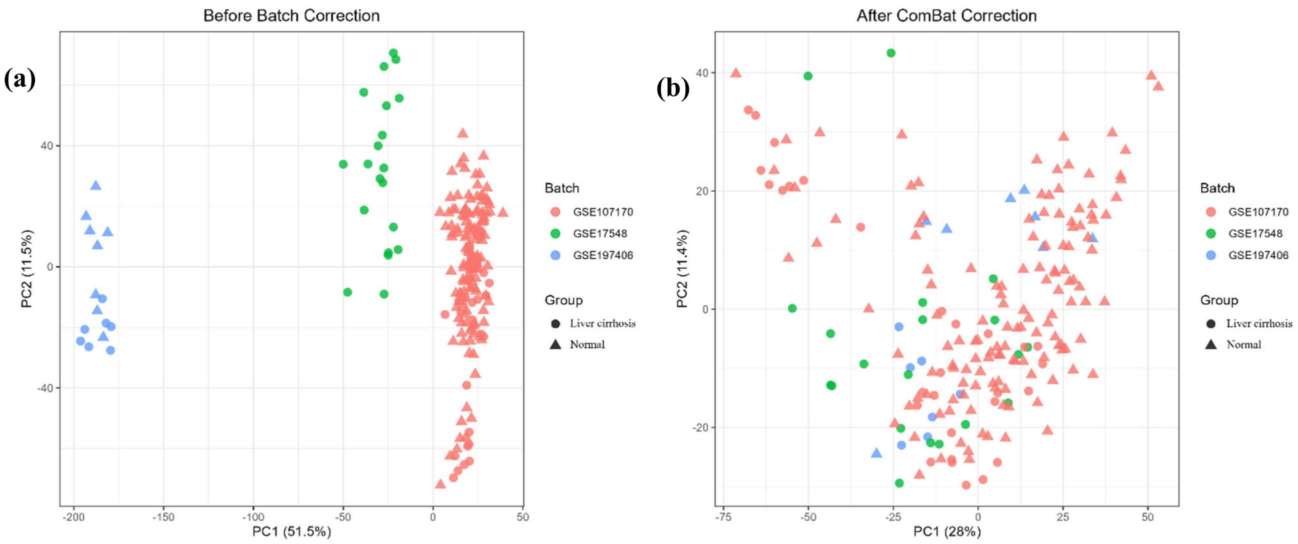

Figure S1 Principal component analysis (PCA) for batch effect assessment and correction. (a) PCA plot of the merged transcriptomic data from three independent datasets (GSE197406, GSE17548, and GSE107170) before batch correction. Samples are colored by their respective dataset of origin, showing significant study-specific clustering. (b) PCA plot after batch effect correction using the ComBat algorithm. The inter-study technical variations were successfully eliminated, with samples from different cohorts intermingling effectively. Points represent individual samples, colors indicate different datasets, and shapes denote clinical status.
